# Risk Stratification on Pheochromocytoma and Paraganglioma from Laboratory and Clinical Medicine

**DOI:** 10.3390/jcm7090242

**Published:** 2018-08-27

**Authors:** Noriko Kimura, Kazuhiro Takekoshi, Mitsuhide Naruse

**Affiliations:** 1 Department of Clinical Research Pathology Division, Department of Diagnostic Pathology, National Hospital Organization Hakodate Hospital, Hakodate 041-8512, Hokkaido, Japan; 2 Division of Sports Medicine, Faculty of Medicine, University of Tsukuba, Tsukuba 305-8575, Japan; k-takemd@md.tsukuba.ac.jp; 3 Department of Endocrinology, Metabolism and Hypertension, National Hospital Organization, Kyoto Medical Center, Kyoto 612-8555, Japan; mnaruse@kyotolan.hosp.go.jp

**Keywords:** pheochromocytoma, paraganglioma, GAPP, metastasis, prognosis, catecholamine, gene mutation, immunohistochemistry, pathology, diagnosis

## Abstract

Pheochromocytoma (PCC) and sympathetic paraganglioma (PGL) are rare neuroendocrine tumors characterized by catecholamine production in the adrenal medulla and extra-adrenal paraganglia. PCC and PGL (PPGL) with metastasis was termed malignant PPGL. However, the distinction between “benign” and “malignant” PPGLs has been debated. Currently, all PPGLs are believed to have some metastatic potential and are assigned malignant tumors (ICD-O/3) by the WHO Classification of Endocrine Organs (2017, 4th edition). Therefore, the previous categories benign and malignant PPGL have been eliminated in favor of risk stratification approach. The Grading of Adrenal Pheochromocytoma and Paraganglioma (GAPP) is a tool for risk stratification for predicting metastasis and the prognosis of patients. At least 30% of PPGLs are hereditary, with 20 genes identified and genotype-phenotype correlations clarified. Of these genes, *VHL, RET* and *NF1* have been well investigated and are the primary cause of bilateral PCC. In addition, mutation of succinate dehydrogenase gene subunits *SDHB* and *SDHD* are strongly correlated with extra-adrenal location, younger age, multiple tumors, metastasis and poor prognosis. Disease stratification by catecholamine phenotype and molecular profiling correlates with histological grading by GAPP. PPGLs should be understood comprehensively based on clinical, biochemical, molecular and pathological data for patient care. A flow chart for pathological diagnosis is included.

## 1. Introduction 

Pheochromocytoma (PCC) and paraganglioma (PGL) are rare neuroendocrine tumors that arise in the adrenal medulla and the extra-adrenal paraganglia, respectively. There are two types of PGL, parasympathetic and sympathetic. Both PCC and sympathetic PGL are catecholamine-producing tumors, which often have a common genetic basis and functional similarities. The incidence of PCC and PGL (PPGLs) is 1–3 individuals per 100,000, with 500–1600 new cases in the United States per year [1,2]. Most PCC present in the fourth to fifth decades of life, with a roughly equal sex distribution. The incidence of PGL is 10–15% that of PCC. PPGLs are characterized by their broad age of incidence, familial association, multifocal tendency and metastasis. At least 30% of PPGLs are hereditary and susceptible genes have been identified [1,2]. A prolonged hypoxic state such as Eisenmenger’s syndrome may be related to PPGL pathogenesis [3]. The risk of metastasis is 10–20% in PCC and up to 50% in PGL depending on the genotype [4,5,6]. The data presented here are primarily regarding PCC and sympathetic PGLs.

Since the beginning age of PPGL research about 50 years ago [7], PPGLs have been classified into either benign or malignant tumors. Despite of much research, there are no universally acceptable histologic criteria predicting metastasis. Thus, histology has been considered useless for differentiating between benign and malignant tumors; malignant PCC was defined only by the presence of metastasis (World Health Organization (WHO), 2004) [8]. The lack of a histological, molecular, or genetic criteria that can absolutely differentiate between benign and malignant PCC/PGL presents an enormous clinical challenge [9]. As a result, the European Clinical Guideline decided that all patients with PPGLs should be followed up for at least 10 years and high-risk patients (young, genetic disease, large tumor and PGL) should be offered lifetime annual follow ups [10]. 

Japan was found to have a similar situation. A nationwide survey of PPGLs determined in 2010 that 2920 patients had PPGLs, 320 of which had metastases [11]. Among patients with 320 malignant PPGL, 37% were initially diagnosed as benign and 60% showed absence of metastasis at initial operation. It was concluded from these results that the diagnosis of benign is not correct for PPGLs with no metastasis at the time of the initial operation and long-term follow up is necessary for patients with PPGL even though the pathologic diagnosis is benign. Based on such clinical data, the WHO Endocrine Tumor Classification 4th edition (2017) [1,2] [decided on a New Concept that all PPGLs have some metastatic potential and assigned an International Classification of Diseases for Oncology (ICD-O)-3 (malignant tumors) for all PPGLs, eliminating the previous categories of benign and malignant tumors in favor of an approach based on risk stratification [1,2]. Risk stratification is required to tailor the follow-up protocol after complete resection of PPGLs [12].

## 2. Pathologic Risk Stratification

To break through the difficulty of pathologic diagnosis of PCC, Thompson used a scoring system, Pheochromocytoma of the Adrenal Gland Scaled Score (PASS), which consists of 12 parameters and scores up to 20 points. Tumors with a PASS score ≥4 were defined as having increased metastatic potential, whereas those with a score <4 were considered not to have metastatic potential [13]. The utility of PASS was validated by seven experienced pathologists that concluded there is significant interobserver and intraobserver variation and they could not currently recommended PASS for clinical prognostication [14]. Kimura et al. [15] then developed another scoring system, Grading of Adrenal Pheochromocytoma and Paraganglioma (GAPP), composed of six parameters that have been considered prognostic factors in many previous reports, including some from PASS and Kimura’s own experience.

### 2.1. GAPP

GAPP is characterized by first evaluating the malignant grade of PCC and sympathetic PGL together, in contrast to PASS, which is only for evaluating PCC. Next, catecholamine phenotypes are analyzed and finally, the malignancy is graded as low risk, intermediate risk, or high risk for metastasis. Thus, predicting patient survival is based on risk grade instead of benign or malignant PPGLs. Histological grading is based on a scoring system composed of six parameters (risk factors for metastasis): Histological pattern, cellularity, comedo necrosis, capsular/vascular invasion, Ki67 labeling index and catecholamine phenotype, with a total of 10 possible points (Table 1). A score of 0–2 is well, 3–6 is moderately and 7–10 is poorly differentiated types (Table 2). Of these PPGLs, approximately 70% are well differentiated, which very rarely metastasize and no patients died of this type of tumor. The well differentiated tumors are compatible with so-called benign PPGLs. In the remaining PPGLs, approximately 20% are moderately and 10% are poorly differentiated, with high metastatic potential (60% and 88%, respectively). The five-year survival rates of the patients are 100%, 67% and 22% for well, moderately and poorly differentiated PPGLs, respectively. Effectively, a GAPP score of 0–2 is low risk, of 3–6 is intermediate risk and of 7–10 is high risk (Table 3). A clinical requirement for risk stratification is urgent as the clinical course of patients with malignant PPGL is remarkably variable and an individualized approach to patients with metastatic PPGL is warranted [16]. Furthermore, risk stratification is required to tailor the follow-up protocol after complete resection of PPGL [12]. GAPP could be utilized for urgent clinical requirements, as Koh et al. have validated GAPP for the prediction of metastatic potentiality [17].

### 2.2. Catecholamine Type and Metastasis

PPGLs are characterized by production of catecholamine. Catecholamines, such as adrenaline, noradrenaline and dopamine, as well as tumor location and ratio of metastasis, are intimately correlated in PPGLs. Adrenaline-producing tumors, those that produce adrenaline only or adrenaline plus noradrenaline, are 100% of adrenal gland origin, with a metastatic ratio of 13%. Noradrenaline-producing PPGLs are those tumors in which noradrenaline only or noradrenaline plus dopamine are produced; 50% of PCCs and 100% of PGLs are of this type. The metastatic ratio is 2 times higher in noradrenaline-type than adrenaline-type tumors in PCCs and approximately 3 times higher in noradrenaline-type tumors in combined PCCs and PGLs (Table 4). Thus, catecholamine type constitutes an important risk factor. Noradrenaline-producing PPGLs lack phenylethanolamine *N*-methyltransferase (PNMT), the enzyme that converts noradrenaline to adrenaline and are considered to be less differentiated than adrenaline-producing tumors based on catecholamine synthesis. In addition, dopamine hypersecretion is considered a feature of immaturity and a marker for malignant PPGLs [18]. Dopamine-producing PPGLs are typically non-functioning; Eisenhofer et al. [19] reported that the plasma level of methoxytyramine, the O-methylated metabolite of dopamine, is 4.7-fold higher in patients with metastases than in those without, suggesting its use as a potential biomarker. The mechanisms underlying non-functioning PPGLs are not fully understood. A deficiency of catecholamine synthesizing enzymes may partially, but not fully, account for this phenomenon [20]. Therefore, the correlation of non-functioning PPGLs and patient prognosis should be further investigated.

## 3. Molecular Risk Stratification

There are 20 susceptibility genes for PPGLs at present: *SDHA*, *SDHAF2*, *SDHC*, *KIF1B*, *TMEM127*, *FH*, *NF1*, *RET*, *VHL*, *SDHD*, *SDHB*, *MAX*, *HRAS*, *ATRX*, *EPAS1/HIF2A*, *MEN1*, *EGLN1/PDH2*, *EGLN2/PDH1*, *MDH2 and IDH1* in various types of mutations including germline only, germline and somatic, somatic only, somatic and somatic mosaicism and single patients or families [21,22]. Germline mutations in predisposition genes are found in 25–30% of PPGLs overall [23]. Germline mutations in succinate dehydrogenase subunit x (*SDHx*) including *SDHA*, *SDHB*, *SDHC*, *SDHD* and *SDHAF2* are the most common genetic cause of PPGLs, occurring in up to 25% of cases [24,25]. The next most commonly associated genes are *VHL* (4–10%), *RET* (1–5%) and *NF1* (1–5%) [26]. PGL1 syndrome (*SDHD*) and PGL2 syndrome (*SDHAF2*) are notable for their high frequency of multifocal tumor development and for parent-of-origin inheritance. PGL4 syndrome (*SDHB*) is notable for an increased risk of malignant PPGLs. PGL3 syndrome (*SDHC*) and PGL5 syndrome (*SDHA*) are less common [26]. Human hereditary paraganglioma pheochromocytoma syndrome (HPPS), including *SDH* types 1–5, is an autosomal dominant disorder characterized by familial predisposition to PPGLs and occasionally combined with renal cell carcinomas, gastrointestinal stromal tumors, neuroendocrine tumors of the pancreas and intestine and rarely, pituitary adenomas [27]. Proportion of gender and age-specific in hereditary PPGL is reported in Table 5 [28].

### 3.1. Immunohistochemistry for Gene Mutations

Loss of SDHB protein immunoreactivity in tumors with *SDHx* mutation is reported with 100% sensitivity and 84% specificity [28]. The following studies validated that SDHB immunohistochemistry can be used for screening of patients with HPPS using paraffin-embedded tumor tissues [29,30,31]. The *SDHB* mutation is the only established factor that indicates future metastasis. Therefore, it is important to perform SDHB immunohistochemistry for all PPGLs except the adrenaline-producing type. Patients with SDHB-negative tumors should be carefully monitored for a long period of time due to the high possibility of metastasis.

In addition to *SDHx* PPGLs, *VHL* tumors are sometimes SDHB-immunohistochemistry negative or stain very weakly. The reasons for such phenomenon may be explained such as tumors associated with *SDH* deficiency display notable upregulation of hypoxia-responsive genes like *VHL* and this is shared by PPGLs associated with mutations in *VHL* [32,33]. In addition, immunohistochemistry for SDHA-negative or MAX-negative tumors indicate an *SDHA*- [34] or *MAX*-mutation [35,36] retrospectively. Therefore, if immunohistochemical analysis is negative for SDHB, SDHA, or MAX, gene analysis should be performed to determine if a mutation is actually present, particularly in younger patients or patients with bilateral, extra-adrenal, or familial PPGLs.

### 3.2. Differential Diagnosis and Risk Stratification

A microarray-gene expression profile demonstrates that Cluster 1 and Cluster 2 clearly differentiate between and correlate with types of genes, signaling pathways and tumor phenotypes [20,23,37,38]. Furthermore, Cluster and GAPP produce very similar results regarding genotype and phenotype correlation (Table 6). The combined data of Cluster and GAPP [15,19,36] may greatly contribute to identifying and selecting the type of therapy and treatment patients receive.

## 4. Flowchart for Differential Diagnosis

**Figure 1:**
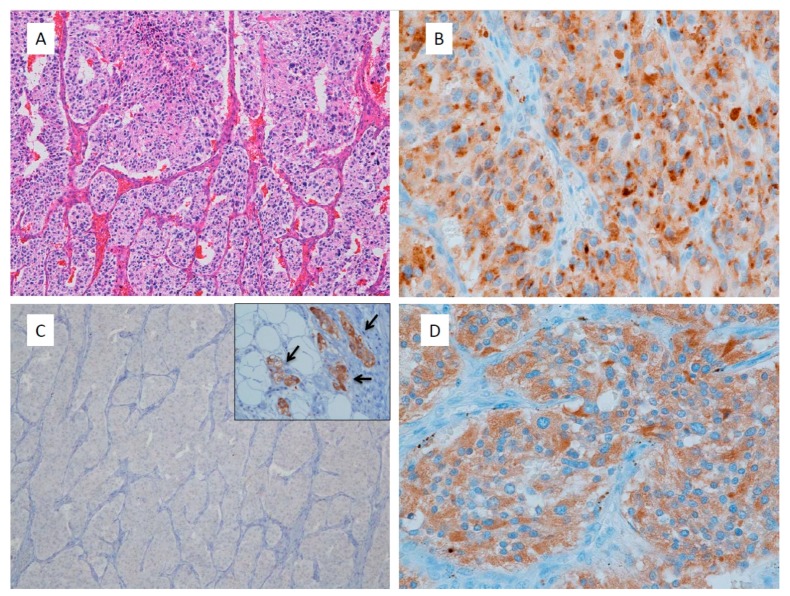
Non-functioning retroperitoneal paraganglioma. (**A**) This tumor shows large irregular zellballen pattern, high cellularity, comedo necrosis, vascular invasion, 14% of Ki67 labelling index and total score of CAPP is 9 points; (**B**) Chromogranin A immunostaining shows dot-like reactivity at Golgi area of tumor cells; (**C**) Tyrosine hydroxylase immunohistochemistry shows negative staining in all tumor cells. Inset is internal control of sympathetic ganglion cells adjacent to the tumor; (**D**) Dopamine beta-hydroxylase immunostaining shows positive reaction in tumor cells even like this non-functioning paraganglioma.

**Figure 2:**
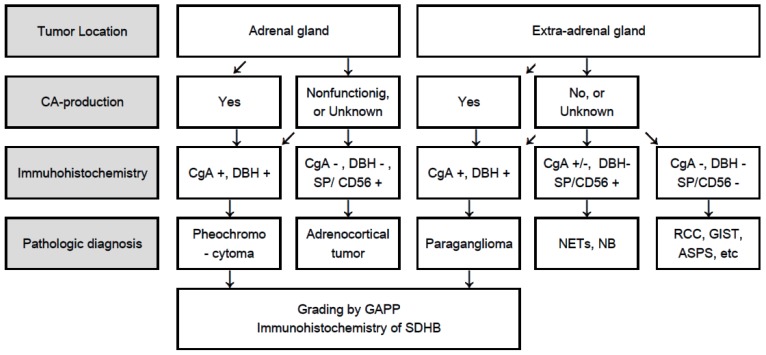
Flowchart for differential diagnosis of PPGLs based on tumor location, catecholamine production, and immunohistochemistry. CA: Catecholamine; CgA: Chromogranin A; DBH: Dopamine β-hydroxylase; SDHB: Succinate dehyrogenase subunit B; PCC: Pheochromocytoma; PGL: Paraganglioma; GAPP: Grading of adrenal pheochromocytoma and paraganglioma; SP: Synaptophysin; CD56: Neural cell adhesion molecule; NETs: Neuroendocrine tumors; NB: Neuroblastoma; RCC: Renal cell carcinoma; GIST: Gastrointestinal stromal tumor; ASPS: Alveolar soft part sarcoma.

Pathological diagnosis of PPGLs is sometimes difficult for general pathologists due to their very low incidence and histological variety, particularly in cases of non-functioning, or catecholamine data are unknown tumors. For example, adrenocortical carcinoma and neuroendocrine tumors are sometimes misdiagnosed as PPGLs. Gastrointestinal stromal tumors, adult neuroblastoma and alveolar soft part sarcoma are rarely needed for differential diagnosis. Immunohistochemistry is the most useful tool for differential diagnosis. All functioning and non-functioning PPGLs express chromogranin A (CgA) and dopamine β-hydroxylase (DBH) [20]. CgA is an essential protein for PPGLs as well as for normal adrenal medulla. Thus, if immunohistochemistry for CgA is negative, PPGLs should be ruled out. Other neuroendocrine markers such as synaptophysin and neural cell adhesion molecule (CD56) are general markers for neuroendocrine tumors besides PPGLs, as well as some adrenocortical tumors. Adrenocortical carcinomas or neuroendocrine tumors of other organs are sometimes misdiagnosed as non-functioning PPGLs. Thus, if only synaptophysin and CD56 are positive, this is not sufficient evidence for a diagnosis of PPGL. Tyrosine hydroxylase (TH) and DBH are essential enzymes for catecholamine synthesis and both antibodies are useful for identification of catecholamine-producing tumors. However, some non-functioning PPGLs including only dopamine-producing or *SDHB*-related PPGLs lack TH immunoreactivity [39,40], but constantly positive for DBH, making DBH a better marker than TH for identification of PPGLs [20,36] (Figure 1). A flowchart for pathologically differential diagnosis of PPGLs is presented (Figure 2).

## 5. Summary and Conclusions

Recent research on PPGLs shows remarkable progress and comprehensive study of clinical, molecular, biochemical and pathological investigations revealed some of the complicated problems associated with PPGLs. Cluster stratification and GAPP intimately correlate which means genotype and phenotype correlation becomes clear in hereditary PPGLs. Immunohistochemistry of SDHB, SDHA, and MAX is a powerful tool for screening these hereditary PPGLs. Catecholamine are specific products in PPGLs and they have been focused for diagnosis and therapy by endocrinology and radiology area. Pathologists should also pay attention to biochemical features of PPGLs to make precise and useful diagnosis for patients. Non-functioning PPGLs including *SDHB* mutation in part but not all are interested in the view point of cell maturation and function of catecholamine synthesis and secretion. Many issues are still remained for further research, in particular, clarifying the mechanism of tumor metastasis and developing a therapy for highly malignant PPGLs in which responsible genes are not yet determined.

## Figures and Tables

**Table 1:** Parameters and score in grading of adrenal pheochromocytoma and paraganglioma (GAPP).

Parameters	Score
**Histological Pattern**	
Zellballen	0
Large and irregular cell nest	1
Pseudorosette (even focal)	1
**Cellularity**	
Low (less than 150 cells/U *)	0
Moderate (150–250 cells/U *)	1
High (more than 250 cells/U *)	2
**Comedo Necrosis**	
Absence	0
Presence	2
**Vascular or Capsular Invasion**	
Absence	0
Presence	1
**Ki67 Labelling Index**	
Less than 1%	0
1–3%	1
More than 3%	2
**Catecholamine Type**	
Adrenaline type (A **, or A + NA ***)	0
Noradrenaline type (NA, or NA + DA ****)	1
Non-functioning type	0
**Total Maximum Score**	10

U *: Cells in Unit of 10 × 10 mm micrometer under high power field (×400); A **: Adrenaline; NE ***: Noradrenaline; DA ****: Dopamine.

**Table 2:** GAPP: Total Score and Grading.

Score	Grading
0–2	Well differentiated type
3–6	Moderately differentiated type
7–10	Poorly differentiated type

**Table 3:** GAPP Score and Risk Stratification.

Total Score (Points)	Histological Grade (Frequency)	Metastatic Rate	5-Year Survival (%)	Risk Stratification
0–2	Well differentiated (68%)	3.6%	100	Low
3–6	Moderately differentiated (22%)	60.0%	66.8	Intermediate
7–10	Poorly differentiated (10%)	88.2%	22.4	High

**Table 4:** Catechoamine types, tumor locations and metastasis.

Catecholamine Types	Number of Patients	Number of Metastasis	Ratio of Metastasis (%)
Epinephrine	78	11	14.1
Norepinephrine	79	29	36.7
(Adrenal)	(49)	(13)	(26.5)
(Extra-adrenal)	(30)	(15)	(50.0)
Non-functioning (Extra-adrenal)	6	1	16.7
Total Number	163	41	25.1

**Table 5:** Proportion of age and gender in hereditary PPGLs with syndromes.

Syndrome	Number	Gene Mutated	Gender (Male/Female)	Age Range (Years; Mean)	Pheochromocytoma	Paraganglioma
NF1	12	*NF1*	3/9	29–67 (44.2)	12	0
MEN2	24	*RET*	8/16	18–76 (35.6)	24	0
VHL	29	*VHL*	12/13 (4 U)	7–62 (25·6)	21 (3 U)	5
PCC-PGL	36	*SDHB*	13/12 (11 U)	10–63 (34.6)	11 (7 U)	18
PCC-PGL	5	*SDHC*	2/3	15–47 (30.6)	0	5
PCC-PGL	61	*SDHD*	25/35 (1 U)	16–72 (40.9)	5 (3 U)	53
Sporadic	53	*None*	17/34 (2 U)	12–79 (49.3)	34 (1 U)	18

NF-1: Neurofibromatosis type 1; MEN2: Multiple endocrine neoplasia type 2; VHL: Von Hippel-Lindau disease; PCC-PGL: Familial paraganglioma-pheochromocytoma syndrome; U: Unknown.

**Table 6:** Correlation of Cluster Stratification and GAPP.

	Cluster 1	Cluster 1	Cluster 2
Gene	*SDH* x *(SDHA*, *B*, *C*,*D*), *SDHAF2*, *HIF2*, *KIF1B*, *PHD2*, *HRAS*, *FH*, *HIF-1*	*VHL* Sporadic Noradrenergic	*RET*, *NF1*, *MAX*, *TMEM127*, Sporadic adrenergic
Signaling pathways	Pseudo hypoxia (HIF-1a) & aberrant VEGF signaling		Kinase signalling: PI3 kinase/AKT, RAS/RAF/ERK, & mTorC1/p70S6K
Catecholamine type	DA, mixed DA & NA	Noradrenaline	Adrenaline
Secretory phenotype	Immature	Immature	Mature
Tumor location	Extra-adrenal	Adrenal & Extra-adrenal	Adrenal
Age of presentation	Early (under 30 year-old)	Early	Late
Metastasis	Frequent	Occasional	Rare
Metastatic risk by GAPP	Intermediate–High	Low–Intermediate	Low

Cluster stratification: Microarray-gene expression profiling; DA: Dopamine; NA: Noradrenaline; A: Adrenaline.
